# Strengthening health system building blocks: configuring post-COVID-19 scenario in Pakistan

**DOI:** 10.1017/S1463423621000128

**Published:** 2021-03-25

**Authors:** Babar Tasneem Shaikh

**Affiliations:** JSI Research & Training Institute Inc., Islamabad 44000, Pakistan

**Keywords:** building blocks, COVID-19, health system, Pakistan, pandemic

## Abstract

**Aim:**

To gauge the level of preparedness of health system of Pakistan in the wake of Corona Virus Disease 2019 (COVID-19) pandemic.

**Background:**

The global COVID-19 outbreak and its subsequent repercussions and implications, after being declared as a pandemic by the World Health Organization (WHO), exposed all the inherent, lingering, and acute shortcomings of the health systems in many developing countries and Pakistan was no exception.

**Methods:**

A detailed literature review was done which included peer-reviewed articles on COVID-19 and health system, published in local and international journals, WHO and World Bank’s publications, and the documents and official reports of the government. Focus was to glean and cite strategies adopted by the developing countries in response to COVID-19 and to see the applicability of those which are feasible for Pakistan.

**Findings:**

Level of preparedness was minimal and the response to manage the outbreak was weak. Based on toll of the cases and number of deaths, this public health threat turned out to be a catastrophe beyond the controlling authority and capacity of the health system, and hence other sectors and agencies had to be engaged for devising a concerted and integrated response to deal with the emergency. Governance was disorderly, financing was inadequate, human resources were not trained, supplies and logistic were not stocked, information system was patchy, and research capacity was limited, and most of all the service delivery was in a biggest chaos of times. COVID-19 demanded to re-configure the health system of Pakistan.

**Conclusion:**

Improving the emergency preparedness of the hospitals is the foremost and an urgent need. A strong national public health system in Pakistan is needed to rapidly investigate and analyze the reports, assess the magnitude of the public health risk, share real-time information, and implement public health control measures in a concerted and systematic demeanor.

## Background

Given the extremely contagious nature of the disease, various attempts are being made by enforcing nationwide smart lockdowns and urging people to practice social distancing and frequent handwashing to minimize transmission. Nevertheless, widespread panic has also led to frustration and exhaustion, causing people to disregard these precautions, which has unfortunately resulted in a possible second wave of the pandemic already (Xu and Li, 2020). This form of corona virus disease has proven to be a difficult one to tackle because of insufficient knowledge on this type of virus, that is, its characteristics, virulence, incubation period, mode of transmission, preventive measures, etc. The ability of any health system to respond to external shocks of different kinds may be one of the key features of resilience (Hanefeld *et al.*, 2018). Corona Virus Disease 2019 (COVID-19) pandemic has revealed the suboptimal resilience of even those health systems which are classified as high-performing (El Bcheraoui *et al.*, 2020). World Health Organization (WHO)’s building blocks for health systems strengthening sound déjà vu in this scenario (World Health Organization, 2007a). International Health Regulations 2005 also suggested critical steps for devising national action plans and strategies for enhancing the level of health system’s preparedness for dealing with pandemics (World Health Organization, 2007b). The rationale of writing this paper is not only to gauge the level of preparedness of health system of Pakistan in the wake of COVID-19 pandemic but also to highlight some of the best practices that can be adopted to make a more responsive health system. For developing this manuscript, a detailed literature review was done from June to August 2020 which included peer-reviewed articles on COVID-19 and health system, published in local and international journals using PubMed/Medline as the database. This part of review was done on eight papers published around COVID-19 and health system. Moreover, WHO and World Bank’s publications and the documents and official reports of the government were also reviewed. Focus was to glean and cite strategies adopted by the developing countries in response to COVID-19 and to see the applicability of those which are feasible for health system of Pakistan.

## Health system’s response in Pakistan

COVID-19 outbreak after being declared as a pandemic by the WHO exposed the inherent, lingering, and acute shortcomings of the health systems in many developing countries and Pakistan was no exception. The likelihood of Pakistan getting overwhelmed with COVID-19 exposure was quite strong because of weak health system, burden of disease, poverty, and malnutrition. In general, the developing countries’ health systems’ machinery struggled to come up with a national-level response to manage the outbreak. Hence, other sectors and agencies such as planning and development, information and broadcasting, education, finance, National Disaster Management Authority, armed forces, development partners, and non-governmental organizations (NGOs) had to be engaged in framing a concerted and integrated response to deal with the crisis. Without a collaborative approach, individual systems in isolation would never have been capable to deal with an outbreak of this magnitude. COVID-19 has come as an eye-opener and it is time to re-configure the health system of Pakistan for saving precious human lives from such preventable causes in the future. Preparedness for making health systems resilient is the key (Legido-Quigley *et al.*, 2020).

## Strengthening building blocks and strengthening the health system

Since health system’s readiness and preparedness are the focus of this paper, therefore, WHO’s health system building blocks framework (World Health Organization, 2007a) is used for developing the case for health system of Pakistan.

### Governance

Governance of any country in the time of external shocks becomes even more critical (Hanfeld *et al.*, 2018). When the health sector is under a calamity or is hit by a catastrophe, good governance becomes even more important to manage the overall affairs and to deal with the menace with a national consensus and a unified strategy. Unfortunately, since the 18^th^ constitutional amendment in 2010 when health as a subject was devolved to the provinces, that is, subnational level in Pakistan, there has been a lack of coordination and a state of tug of war between the federation and the provinces, especially where there is a different political party on the treasury benches (Mazhar and Shaikh, 2016). In the case of COVID-19 crisis, the same sort of distrust, allegations, and lack of coordination was seen between the federation and the provinces regarding decision-making, planning, execution, service delivery, information sharing, and monitoring.

In the post-COVID-19 times, it would be imperative to figure out what type of governance suits health sector the best. Policy, strategy, action planning, service delivery, and even monitoring are devolved to the provincial level; however, regulation and coordination are two functions still with the federal government (Nishtar, 2011). Amidst the health system challenges, political will for timely, coordinated and collaborative actions can strengthen the resilience and response of the health system during a health emergency (Hunte *et al.*, 2020). There needs to be a national review and consensus on the implications of the devolution of health to the provinces. Perhaps it is time to re-think for a better governance model for health system to face such national crises in the future.

### Financing

The health budget has always been meager in Pakistan and since the last many years got a consistently low allocation of below 1% of the total gross domestic product; most of which is skewed toward the nondevelopment or recurrent side (Ahmed and Shaikh, 2008; Ministry of Finance, 2019). Public sector health system, therefore, has struggled to deliver an optimal quality primary care to the people of Pakistan. Private sector hence filled the void and operated in the country in the free unregulated market, largely for profit. As a result, major proportion of expenditure on health (around two-third) is private, that is, out of pocket (World Bank, 2020). Furthermore, after the 18^th^ constitutional amendment and devolution of health as a provincial subject, the federal share of health budget has shrunk even more and it is the responsibility of the provincial governments to ascertain the need and allocate the appropriate share of budget to health sector. At the onset of COVID-19 crisis, with a very narrow fiscal space for health, Government of Pakistan could allocate only USD0.5 million as an initial response. However, for the overall national action plan covering the needs of surveillance, rapid response teams, equipment/supplies for diagnostic labs, health and quarantine facilities, personal protective equipment, strengthening of points of entry, etc., it was estimated to cost around USD11 million (Ministry of National Health Services, Regulations & Coordination, 2020a). Development partners and donors came forward with financial and logistics assistance, among which World Bank, Asian Development Bank, United States Agency for International Development (USAID), Department for International Development (DFID), and others pitched in. Amidst the crisis, the provincial governments could not figure out as to how to mobilize extra funds to deal with the emergency of such scale, and the realization was quite soon evident that the federal government will have to bail them out.

It would be very opportune to re-think the financing of health care in this country; both for normal and emergency times. In a national crisis, donors do come forward to fill the gap, but this aid has multiple strings attached and of course is not sustainable. A new model of foreign assistance for health, one that is focused on stronger and more resilient health systems, should be the top priority of development partners. The overall budget envelop for health in Pakistan ought to be increased, disciplined, and judiciously spent on the welfare of the people. With the lessons learned from COVID-19, policy-makers in the health system of Pakistan must realize the repercussions of chronic under-spending in health the dire need urgently expanding the budgetary space for health. Thoughtful financing reform would be required in the post-COVID times to put the health system of Pakistan on a journey of self-reliance (Shaikh and Ali, 2020).

### Human resources

The health workforce is crucial to configure a health system’s response to shocks, but if not trained to deal with infectious disease emergencies, they can be the most vulnerable individuals (Gostin and Friedman, 2015). A specialized training is therefore needed to protect their patients as well as themselves from cross-infections. With the outburst of COVID-19 in Pakistan, not only numbers of health care providers were found to be low, but there were issues with their distribution, place of postings, and concentration in major urban centers of the country. The physician population ratio in Pakistan is one of the lowest in the region, that is, 0.96/1000, so is the case for nurse population ration, that is, 0.49/1000 (Ministry of National Health Services, Regulations & Coordination, 2018). Most of these were known facts and the health system was lingering on in this dim and grim situation. With the emergence of COVID-19, not only these issues were re-ignited but it was immediately realized that the health personnel are not competent to deal with new infections. There were no trained rapid response teams at provincial, district, or subdistrict levels having a capacity for active case detection and management, surveillance, and data management and analysis, etc. Frontline health providers were in need of refresher training on the use of ventilators, infection prevention and control, correct use of personal protective equipment, and home-based care for COVID-19 patients with mild symptoms. Again, the government had to rely on donors and other technical agencies for conducting the capacity building for various cadres of health staff.

Human resources in the health sector would require a continuing education program to keep their skills set at par with the epidemiological and demographic transition in the country. Similarly, there is a need for a national human resource strategy to build their core capacity to fulfill the commitments of International Health Regulations and global health security to which Pakistan is a signatory.

### Medicines, supplies, and logistics

COVID-19 is not treated with any specific drugs, and only antipyretics, anti-inflammatory, and antihistamines may be needed to give a transitionary relief to the patient with mild symptoms of COVID-19. In cases with moderate symptoms, few antibiotics may be required. Only severe cases (around 1%) with co-morbidities and the elderly would require hospitalization, intensive care, and at times the ventilators for mechanical ventilation (Ministry of National Health Services, Regulations & Coordination, 2020b). During the planning phase for dealing with COVID-19, it was revealed that the provinces do not have sufficient emergency stock of medicines and other medical supplies such as personal protective equipment, even simple surgical masks, and logistics, that is, extra hospital beds, oxygen, ventilators, etc. In the past, NGOs, charitable organizations and relief agencies have lent a hand to the government for arranging all these supplies and logistics. One such instance was the earthquake of 2005 in Pakistan. Such breaches in the health sector have become raison d’être for NGOs in the country to plug in the gaps in their expertise and scope of work (Ejaz *et al.*, 2011). During the COVID-19 pandemic, drugs were late in delivery and short in supply due to logistics interruptions and production disruptions because of lockdowns in the country. International trade was also stopped because of the potential transmission of the infection across the borders (Ying *et al.*, 2020). All essential medicines, masks, and oxygen cylinders disappeared from the market or were available at exorbitantly high prices, again a manifestation of an unregulated private health market. Furthermore, government’s own system of procurement is so cumbersome that National Disaster Management Authority had to be called in for initiating the emergency procurement of testing kits, masks, personal protective equipment, and ventilators from the international market. Donors were mobilized to pledge money for purchasing these goods, along with the government’s own money diverted from other development budget pots for the purpose.

The way forward is to establish a national-level health resource management system which should give a consolidated picture of all medicines stocks, supplies, and logistics available throughout the country. Such database is indispensable for planning, resource allocation, procurement, distribution and above all, judicious utilization of the same in the time of medical emergencies of such scale.

### Information system

An integrated, reliable, and composite information system is the backbone of any health system and a must conduit for a disease surveillance system (Baig and Shaikh, 2012). Foremost pre-requisites of an information system include (a) a public health surveillance system which should be comprised of disease surveillance units at the districts, provinces, and federal levels well connected with each other for reporting, monitoring, resource sharing, and decision-making, (b) a legal framework for notifying all communicable diseases and other public health threats, and (c) capacity building of staff of disease surveillance units in case definitions, identification, data analysis, and reporting (World Bank, 2005).

COVID-19 cases upsurge since March 2020 called for an information system or a national dashboard to monitor the progress of infection progression in the country. It was soon realized that there is no single reliable system to depict such authentic data from the subdistricts level to the National Command & Operations Center established to monitor COVID-19 and to make a national unified strategy. Exception was the polio monitoring sentinel sites throughout the country and the workforce whose primary mandate is to collect data on polio. They were eventually given this additional responsibility of collecting and reporting the data on COVID-19 suspected and positive cases. Once again, with the donor’s support, the dormant provincial disease surveillance and response units were strengthened and the district disease surveillance units were established with notification of the rapid response teams in each district for reporting on COVID-19 cases. Since each province has a list of notifiable infectious diseases, it would necessitate to provide the operational budget and logistic support to these disease surveillance units to fulfill their mandate. It would be equally important to build the capacity of the rapid response teams for timely and accurate reporting, so that an integrated disease surveillance system can become a reliable source for decision-making for the identification of hot spots, for smart lockdowns and for other decisions to be taken by the provincial health authorities. The crux of the information system should be contact tracing. When systematically applied, contact tracing will break the chains of transmission of an infectious disease and is thus an essential public health tool for controlling infectious disease outbreaks (World Health Organization, 2020).

### Service delivery

Infectious diseases require two types of services: preventive including vaccination, health education, and raising awareness on hygiene principles and about self-care, and secondly curative services for treatment of the disease. Effective service delivery is not possible with resource constraints, shortage of trained human resources, modern functional equipment, and an overall enabling environment in the health care facilities (Oliveira-Cruz *et al.*, 2003). This one block of health system for its optimal functioning is dependent on all the other system building blocks. Weaknesses of the public sector result in its low utilization, and consequently the private sector flourishes and establishes its for-profit business, which is more focused on curative services and does not find any incentive in providing or promoting the primary preventive care (Shaikh, 2015). COVID-19 cases in Pakistan needed hospital care, although a very small proportion in the beginning of March and April 2020, but gradually the number increased and by the end of May, the capacity of the hospitals was over-flooded. Preparedness of the hospital to deal with the COVID-19 cases was one of the major issues. Isolation wards and high dependency units were nonexistent in many secondary and tertiary care hospitals, staff was not trained to work in such units, beds were insufficient, more than 500 out of total 1800 ventilators were found out of order, and moreover the supplies were depleted. Again, the private sector plugged in the gaps but at a very high cost that many had to pay out of pocket. On the preventive side, primary care facilities and community health workers were not trained in propagating messages on COVID-19 prevention, personal hygiene, social distancing, home-based care, etc. Besides, the routine services got disrupted; and the most important one was polio eradication campaign because of the direct engagement of the polio staff and community health workers in COVID-19 activities.

Health service delivery system must develop a mechanism to absorb epidemic and pandemic shocks and avert possible disruption of basic essential health services during the crisis (Kruk *et al.*, 2016). The system would need to be geared up by establishing quarantines, isolation wards, and high dependency units at least in all secondary and tertiary care public hospitals. Staff would need capacity building on operating the ventilators, rotations in intensive care units, and infection prevention and control. Stocks of supplies and medicines for emergencies and epidemics ought to be kept in each health facility for a buffer period until the new procurement sets in. Service delivery standards, protocols, and standard operating procedures have to be implemented and institutionalized to deliver quality care. This will build the trust and confidence of the people in public hospitals and would save their hard-earned money unreasonably spent in seeking private health care.

## Conclusion

Good governance with interprovincial harmony, increasing fiscal space for health, investing in human resources for health, establishing an inventory management system, promoting the use of information for decision-making, and tailoring service delivery by recognizing the local context and wider systems ‘values’ are essential elements to strengthen the health system. An overarching political will is needed to ensure whether interventions in each block are succeeding. Well-integrated and locally grounded health system may be more resilient to external shocks like COVID-19.
